# Tumor necrosis factor regulates leukocyte recruitment but not bacterial persistence during *Staphylococcus aureus* craniotomy infection

**DOI:** 10.1186/s12974-024-03174-9

**Published:** 2024-07-23

**Authors:** Zachary Van Roy, Tammy Kielian

**Affiliations:** Department of Pathology, Microbiology, and Immunology, University of Nebraska Medical Center, 985900 Nebraska Medical Center, Omaha, NE 68198-5900 USA

**Keywords:** Craniotomy, Biofilm, *S. aureus*, Granulocytes, Macrophage, TNF, Lymphotoxin, TNFR1, TNFR2

## Abstract

**Background:**

Craniotomy is a common neurosurgery used to treat intracranial pathologies. Nearly 5% of the 14 million craniotomies performed worldwide each year become infected, most often with *Staphylococcus aureus* (*S. aureus*), which forms a biofilm on the surface of the resected bone segment to establish a chronic infection that is recalcitrant to antibiotics and immune-mediated clearance. Tumor necrosis factor (TNF), a prototypical proinflammatory cytokine, has been implicated in generating protective immunity to various infections. Although TNF is elevated during *S. aureus* craniotomy infection, its functional importance in regulating disease pathogenesis has not been explored.

**Methods:**

A mouse model of *S. aureus* craniotomy infection was used to investigate the functional importance of TNF signaling using TNF, TNFR1, and TNFR2 knockout (KO) mice by quantifying bacterial burden, immune infiltrates, inflammatory mediators, and transcriptional changes by RNA-seq. Complementary experiments examined neutrophil extracellular trap formation, leukocyte apoptosis, phagocytosis, and bactericidal activity.

**Results:**

TNF transiently regulated neutrophil and granulocytic myeloid-derived suppressor cell recruitment to the brain, subcutaneous galea, and bone flap as evident by significant reductions in both cell types between days 7 to 14 post-infection coinciding with significant decreases in several chemokines, which recovered to wild type levels by day 28. Despite these defects, bacterial burdens were similar in TNF KO and WT mice. RNA-seq revealed enhanced lymphotoxin-α (*Lta*) expression in TNF KO granulocytes. Since both TNF and LTα signal through TNFR1 and TNFR2, KO mice for each receptor were examined to assess potential redundancy; however, neither strain had any impact on *S. aureus* burden. In vitro studies revealed that TNF loss selectively altered macrophage responses to *S. aureus* since TNF KO macrophages displayed significant reductions in phagocytosis, apoptosis, IL-6 production, and bactericidal activity in response to live *S. aureus*, whereas granulocytes were not affected.

**Conclusion:**

These findings implicate TNF in modulating granulocyte recruitment during acute craniotomy infection via secondary effects on chemokine production and identify macrophages as a key cellular target of TNF action. However, the lack of changes in bacterial burden in TNF KO animals suggests the involvement of additional signals that dictate *S. aureus* pathogenesis during craniotomy infection.

**Supplementary Information:**

The online version contains supplementary material available at 10.1186/s12974-024-03174-9.

## Introduction

Access to the intracranial compartment is achieved through craniotomy, wherein a piece of the skull is temporarily removed for numerous indications, including brain tumor excision, hematoma evacuation, or implantation of a medical device [1]. In 3–5% of cases, surgical site infection complicates patient recovery and over half of craniotomy infections are caused by *Staphylococcus aureus* (*S. aureus*), which forms a biofilm on the surface of the bone flap that evades immune and antibiotic clearance, progressing to chronic infection [1, 2]. Therefore, craniotomy infections require an additional surgery to remove the biofilm nidus (i.e., bone flap) and long-term antimicrobial therapy to eliminate residual planktonic bacteria, underscoring the severity of these infections [3–6]. Our previous work has demonstrated compartmentalization of the immune response during craniotomy infection, with adaptive immune cells and monocytes preferentially homing to the infected brain parenchyma. In contrast, the galea and bone flap are dominated by granulocytic myeloid-derived suppressor cells (G-MDSCs) and neutrophils (PMNs) that are also present in the brain, albeit at a lower frequency [1, 7–10]. Both granulocyte populations acquire anti-inflammatory activity within the biofilm milieu by their ability to inhibit T cell proliferation and robust IL-10 production [7, 9]. However, proinflammatory responses are also elicited during *S. aureus* craniotomy infection, as demonstrated by the importance of TLR2- and caspase-1-dependent pathways for bacterial containment [11]. Therefore, it is important to understand the relative contributions of pro- vs. anti-inflammatory cytokines for dictating craniotomy infection outcome with the goal of leveraging this information to augment the former, in combination with antibiotics, to clear infection without the need for additional surgical procedures.

Tumor necrosis factor (TNF) was one of the first cytokines to be described and is a prototypical proinflammatory mediator [12]. Although the cytokine was previously renamed TNF-α in 1985 when lymphotoxin-β was identified and referred to as TNF-β, the discovery of lymphotoxins-α and -β in 1993 demonstrated that there is only one form of TNF [13]; therefore, we refer to the cytokine as TNF throughout the manuscript. TNF is produced by a wide range of immune and parenchymal cells within minutes following injury or infection [14, 15]. Consequently, TNF is tightly regulated at the transcriptional, translational, and post-translational levels [12, 14] and signals through one of two non-redundant cell surface receptors, namely TNFR1 and TNFR2. TNFR1 is constitutively and broadly expressed in most cell types [12, 14, 16] and is responsible for the majority of reported effects of TNF in the literature [16], where receptor binding elicits mitogen-activated protein kinase (MAPK) and NF-κB activation. TNFR2 expression is restricted to leukocytes and a limited number of additional cell types (i.e., endothelial cells) and is important for modulating neutrophil antimicrobial activity [17]. TNF signaling through both receptors elicits pleiotropic effects, including NADPH oxidase and NLRP3 inflammasome priming in PMNs [1, 11], increased blood-brain barrier (BBB) permeability during neuroinflammation [1, 18], generation and activation of myeloid-derived suppressor cells (MDSCs) [19–21], and promoting T cell activation [22]. In terms of *S. aureus* infection, TNF deficiency has been reported to worsen a wide range of diseases, including sepsis [23], brain abscesses [24, 25], arthroplasty [26], and skin abscesses [17]. The protective nature of TNF against infection is also apparent in humans, as TNF polymorphisms [27] and the use of TNF-neutralizing biologics are associated with an increased risk of infection in patients [28–30], including *S. aureus* [22].

Although TNF influences the function of all major immune cell populations that respond to craniotomy infection (i.e., PMNs, G-MDSCs, monocytes, macrophages, microglia, and CD4^+^ T cells), the importance of TNF during CNS biofilm infection has not yet been examined. Here, we show that TNF is critical for stimulating chemokine production and leukocyte recruitment during acute craniotomy infection since both were significantly reduced in TNF KO mice. In addition, loss of TNF in macrophages and TNFR1 or TNFR2 deficiency in granulocytes affected phagocytosis, bacterial killing, and apoptosis in response to *S. aureus*. Despite these phenotypes, the loss of TNF or its receptors did not dramatically alter bacterial burden in vivo, suggesting either the involvement of other inflammatory mediators and/or redundancy in the TNF signaling pathway. The latter possibility was supported by the finding that lymphotoxin-α (*Lta*) expression was significantly elevated in granulocytes from TNF KO mice. Collectively, these findings suggest that TNF contributes to coordinating the immune response to craniotomy infection but that other inflammatory mediators are also involved.

## Materials and methods

### Mouse model of *S. aureus* craniotomy infection

Craniotomy infection was established in 8–10 week-old male and female C57BL/6J wild type (WT; RRID: IMSR_JAX:002365), *Tnf*^−/−^ (TNF KO; RRID: IMSR_JAX:00300), *Tnfr1*^−/−^ (TNFR1 KO; RRID: IMSR_JAX:003242), *Tnfr2*^−/−^ (TNFR2 KO; RRID: IMSR_JAX:002620), or *Pad4*^−/−^ (PAD4 KO; RRID: IMSR_JAX:030315) mice as previously described [2, 7, 10, 11]. Briefly, a surgical plane of anesthesia was achieved by i.p. ketamine and xylazine administration before exposing the cranium with a midline scalp incision. A pneumatic surgical drill (Stryker Corporation) was used to produce a 3–5 mm bone flap on the left side of the skull that was immediately incubated with 10^6^ CFU of the methicillin-resistant *Staphylococcus aureus* (MRSA) strain USA300 LAC13C [31] diluted in 500 µL brain-heart infusion broth for 5 min at 37 °C, which resulted in an infectious inoculum of 10^3^ CFU. Bone flaps were extensively rinsed in 1X PBS to eliminate bacterial broth, blotted to remove excess fluid, and reimplanted. The incision was sutured closed, and animals were monitored under external heat until regaining ambulation and once per day thereafter until euthanized. In some experiments, mice received systemic antibiotics. Briefly, an antibiotic cocktail [25 mg/kg Rifampin (Mylan Institutional; Morgantown, WV) + 5 mg/kg Daptomycin (Camber Pharmaceuticals; Piscataway, NJ) in 1X PBS] was administered via i.p. injection (200–250 µL) starting on day 3 post-infection and every 24 h thereafter until experiment conclusion. Control animals received i.p. injections of an equivalent volume of sterile 1X PBS. The craniotomy infection model is associated with a low mortality rate (< 1%), which is primarily due to complications from anesthesia, and no overt differences in animal appearance were observed between WT mice and any of the KO strains examined.

### Tissue harvesting and processing

Mice were euthanized by isoflurane overdose at days 7, 14, or 28 post-infection. Following perfusion with 1X PBS to clear leukocytes from the vasculature, the bone flap, subcutaneous galea tissue, and infected brain hemisphere were removed and placed in 10% fetal bovine serum (FBS) in PBS. Tissues were then processed separately to obtain single-cell suspensions. Bone flaps were initially vortexed for 30 s to dislodge adherent immune cells, and an aliquot of this suspension was saved for flow cytometry analysis. The remaining bone flap samples were sonicated for 5 min to disperse biofilm, serially diluted, and plated on tryptic soy agar plates supplemented with 5% sheep blood for bacterial enumeration. Galea samples were disrupted with the blunt end of a 5 mL syringe plunger, passed through a filter top FACS tube, and saved for flow cytometry. Brain samples were homogenized through a 70 μm strainer and digested with collagenase IV and DNase I for 15 min at 37 °C before enzyme inactivation with the addition of FBS. Myelin was removed using a 25% Percoll solution by centrifugation for 20 min at 520x*g* with no brake. The resulting cell pellet was washed in PBS, passed through a filter top FACS tube, and saved for flow cytometry analysis. Aliquots of galea and brain were taken immediately following homogenization for bacterial enumeration. Likewise, where indicated, supernatant was collected from pelleted homogenates for subsequent inflammatory mediator analysis or albumin ELISA.

### Quantification of immune infiltrates and intracellular cytokine staining

Single-cell suspensions from the bone flap, galea, and brain were first incubated with TruStain FcX (Cat #101320; BioLegend) before cell surface marker staining with the following antibody panel: CD45-PE/Cyanine7 (RRID: AB_312979), Ly6G-Pacific Blue (RRID: AB_2251161), Ly6C-PerCP-Cy5.5 (RRID: AB_1727558), CD11b-AlexaFluor^®^ 700 (RRID: AB_493705), F4/80-Brilliant Violet 510™ (RRID: AB_2562622), CX3CR1-Brilliant Violet 785™ (RRID: AB_2565938), CD3-BUV737 (RRID: AB_2871136), CD4-Brilliant Violet 650™ (RRID: AB_2562529), CD8-Brilliant Violet 605™ (RRID: AB_2562609), and NK1.1-APC/Cyanine7 (RRID: AB_830871). For TNFR1 and TNFR2 KO experiments, the following adjustments were made to the panel: MHC-II (I-A/I-E)-Brilliant Violet 605™ (RRID: AB_2565894) was included, CD8 was omitted, and Ly6C-FITC (RRID: AB_1186135) was used instead of PerCP-Cy5.5. Due to the lack of adaptive immune cells and microglia in the galea and bone flap, CX3CR1, CD3, CD4, CD8, and NK1.1 antibodies were omitted from these samples. Dead cells were identified and excluded from analysis using a Zombie UV Fixable viability kit (BioLegend; Cat #423108) and Spherotech AccuCount beads (Cat #ACBP-100-10; 8.0–12.9 μm) were added to each sample to enable reporting of absolute cell counts. All acquisition was completed on a BD LSR II-G cytometer with analysis using FlowJo and the gating strategies depicted in Additional File 1: Fig. S1.

When indicated, intracellular staining for TNF was conducted using the Cyto-Fast™ Fix/Perm Buffer Set (BioLegend; Cat# 426803). Briefly, cells from the brain, galea, and bone flap were incubated with 5 µg/mL brefeldin A immediately ex vivo for 4 h. Samples were then surface stained as described above, fixed, and incubated with TNF-PerCP/Cy5.5 (RRID: AB_961434) using Cyto-Fast™ reagents according to the manufacturer’s protocol.

### Quantification of cytokine and chemokine expression in tissue homogenates and cell supernatants

Brain and galea supernatants were collected as described above and analyzed for inflammatory mediator expression using a MILLIPLEX MAP Mouse Cytokine/Chemokine Magnetic Bead Panel (Cat #HT17MG-14 K-PX25, Millipore Sigma). Homogenates were first centrifuged at 14,000 rpm for 10 min at 4 °C to remove residual cellular material and assayed according to the manufacturer’s instructions. A MAGPIX^®^ xMAP instrument (Luminex) was used for analysis with Belysa^®^ Analyst software (Millipore Sigma), and values were corrected for total protein content via BCA assay.

Murine IFN-ɣ, IL-6, IL-10, IL-12, CCL2, and TNF levels were quantified in cell-conditioned medium using a Mouse Inflammation Cytometric Bead Array (Cat #552364, BD Biosciences). IL-12 production was minimal under the conditions tested in this study, and IFN-ɣ is not produced by the innate immune cell populations examined, so neither mediator was reported.

### Generation of primary immune cells

All primary immune cells were prepared from WT, TNF KO, TNFR1 KO, or TNFR2 KO mice. Macrophages and G-MDSCs were derived from the bone marrow of 8- to 12-week-old animals as previously described [32, 33]. Briefly, bone marrow was extracted and filtered with a 70 μm cell strainer prior to red blood cell lysis in sterile H_2_O, followed by rapid correction to isotonic conditions. Macrophages were cultured in RPMI-1640 with 10% FBS, penicillin/streptomycin/fungizone, 2 mM L-glutamine, and 10% conditioned medium from L929 fibroblasts as a source of macrophage colony-stimulating factor (M-CSF) at 37 °C, 5% CO_2_. Medium was replaced on days 3 and 5 with cells used on day 7 in vitro. G-MDSCs were expanded from bone marrow in RPMI-1640 supplemented with 10% FBS, penicillin/streptomycin/fungizone, granulocyte-macrophage colony-stimulating factor (GM-CSF) and granulocyte colony-stimulating factor (G-CSF) (40 ng/mL each) for 3 days at 37 °C with 5% CO_2_, whereupon IL-6 (40 ng/mL) was added. Cells were isolated from the resulting mixed cell population on day 4 in vitro using Ly6G magnetic beads (Miltenyi Biotec). Neutrophils were isolated on the day of use from bone marrow with Ly6G magnetic beads following red blood cell lysis.

### Assessments of apoptosis, phagocytosis, bactericidal activity, and neutrophil extracellular trap (NET) formation

Apoptosis was analyzed using a flow cytometry approach. Leukocytes were added to a 96-well plate at a density of 2 × 10^5^ cells per well and exposed to live *S. aureus* at a 10:1 multiplicity of infection (MOI; bacteria: leukocyte) for 1 h prior to staining with 400 nM Apotracker Green (Biolegend; Cat #427402) for 30 min at 37 °C. Dead cells were identified using a Zombie UV Fixable viability kit (BioLegend). Leukocytes were distinguished from bacteria and cell debris by forward and side scatter gating. Analysis was performed on a BD LSR-IIG cytometer and quantified by FlowJo.

Phagocytosis was assessed by incubating leukocytes with either live *S. aureus*-GFP or *S. aureus*-dsRED strains [34] at a 10:1 MOI for 1 h. Dead cells were excluded, and leukocytes were distinguished from extracellular bacteria and cell debris via forward and side scatter gating. Analysis was performed on a BD LSR-IIG cytometer and quantified by FlowJo.

Bactericidal activity was assessed by gentamicin protection assays as previously described [35]. Cells were seeded in a 96-well plate (2 × 10^5^ cells per well) prior to live *S. aureus* exposure at a 10:1 MOI for 1 h at 37 °C. Following incubation, extracellular bacteria were eliminated by high-dose gentamicin (100 µg/mL) treatment for 30 min at 37 °C. Cells were then washed and incubated in RPMI-1640 medium supplemented with a gentamicin maintenance dose (1 µg/mL) to prevent *S. aureus* outgrowth. At the indicated time points, cells were lysed in H_2_O for 15 min, serially diluted, and plated on blood agar to enumerate intracellular bacteria. Importantly, both gentamicin protection assays and phagocytosis experiments utilized identical bacterial incubation periods (1 h) to permit the interpretation of bactericidal activity along with any changes in bacterial uptake, which were minimal.

For assessing NET formation, bone marrow PMNs were plated in 8-well glass chamber slides at 4 × 10^5^ cells/well and exposed to live *S. aureus*-dsRed at a MOI of 10:1 for 1 h at 37 °C. Cells were then stained with 5 µM Sytox Green (Invitrogen; Cat #S7020) to assess NET formation and imaged using a confocal laser scanning microscope (Zeiss 710) with a 40× oil objective. NETs were discriminated from dead PMNs based on differences in cell size, shape, and transparency of Sytox Green staining characteristic of extracellular DNA release [7, 36, 37] and are reported as the percentage of total cells in each field of view. Representative Z-stack images were taken (1 μm slices) to illustrate NET formation.

### Assessment of blood-brain barrier permeability

Blood-brain barrier (BBB) integrity was assessed using an albumin ELISA as described elsewhere [38–40]. Briefly, supernatants collected from brain homogenates of mice following vascular perfusion were centrifuged to remove residual cellular debris and assayed using a mouse Albumin ELISA Kit (Fortis Life Sciences; Cat# E99-134) according to the manufacturer’s protocol. Data was normalized to total protein content via BCA assay.

### RNA sequencing experiments

Single-cell suspensions were prepared from the galea of WT and TNF KO mice at day 7 post-infection as described above, whereupon live CD45^+^Ly6G^+^ granulocytes were purified by fluorescence-activated cell sorting (FACS). A total of 9 mice were infected for each group (WT and TNF KO) and galea tissues were pooled from 3 mice for a total of 3 biological replicates. RNA was isolated using a Quick-RNA Microprep Kit (Zymo Research; Cat #R1050), and RNA sequencing libraries were prepared with a target of 50 ng RNA per sample. Libraries were sequenced on a NextSeq500 instrument (Illumina) with mid-output 150 cycles (75 paired-end reads) at a sequencing depth of ~ 10 million paired reads per sample. All subsequent data processing and analysis were conducted with Partek Flow Genomics Suite (RRID: SCR_011860). Reads were quality checked and trimmed before alignment to the mouse genome. Additional post-alignment quality control steps were executed to remove features with low read counts, and data was normalized by counts per million plus 1 × 10^− 4^. A gene-specific analysis (GSA) approach was used to determine the optimal differential expression model based on lowest Akaike Information Criterion correction (AICc) and to control for false discovery rate, as described elsewhere [41, 42]. Genes with *p* < 0.05 were considered differentially expressed. Gene set enrichment analysis (GSEA) was performed in Partek Flow to identify differentially regulated pathways with Ingenuity Pathway Analysis (IPA; Qiagen) used as a secondary modality to explore differentially expressed pathways. The resulting dataset has been deposited in the GEO database (GSE252481).

### Statistics

Significant differences between groups for immune populations, inflammatory mediator expression, ELISA, or other assays were determined using either an unpaired two-tailed Student *t*-test, multiple unpaired *t*-test, or two-way ANOVA with Šidák multiple comparisons test in GraphPad Prism (RRID: SCR_002798). Outliers were removed only for Milliplex assays using the ROUT method [43] (with Q = 1%; 0–2 observations per condition) in GraphPad Prism. A *p*-value of < 0.05 was used to reflect statistical significance.

## Results

### TNF mediates granulocyte recruitment during *S. aureus* craniotomy infection

**Fig. 1:**
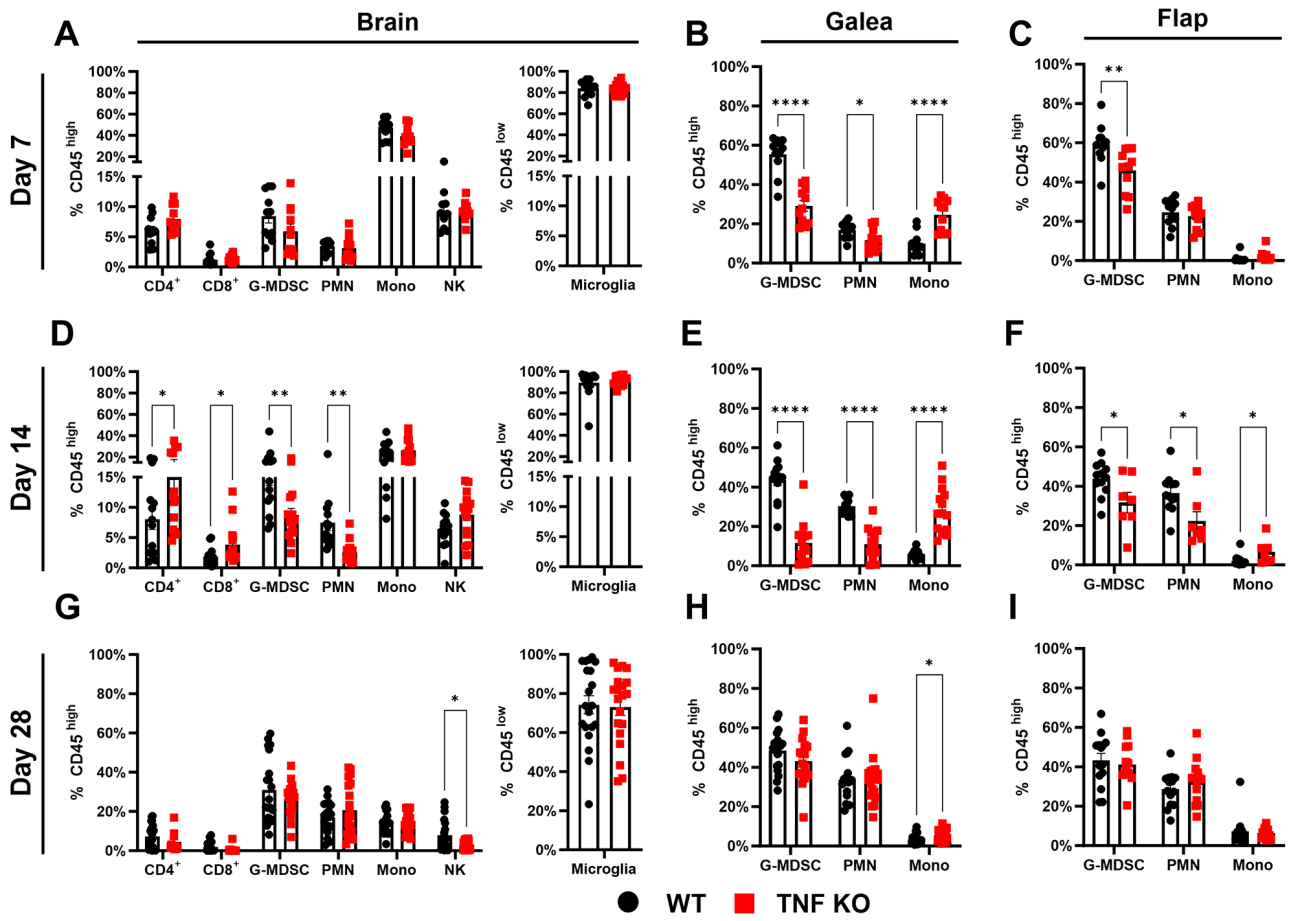
TNF modulates granulocyte recruitment during craniotomy infection. TNF knockout (KO; *n* = 7–35) and wild type (WT; *n* = 11–35) mice were subjected to craniotomy infection and sacrificed at days 7 (**A-C**), 14 (**D-F**), and 28 (**G-I**) post-infection, whereupon immune infiltrates were quantified from the brain (**A**,** D**,** G**), galea (**B**,** E**,** H**), and bone flap (**C**,** F**,** I**). Data was pooled from 2–5 independent experiments (mean ± SEM) and analyzed by multiple unpaired t-test. Mono, monocyte; NK, NK cell; *, *p* > 0.05; **, *p* > 0.01; ****, *p* > 0.0001

**Fig. 2:**
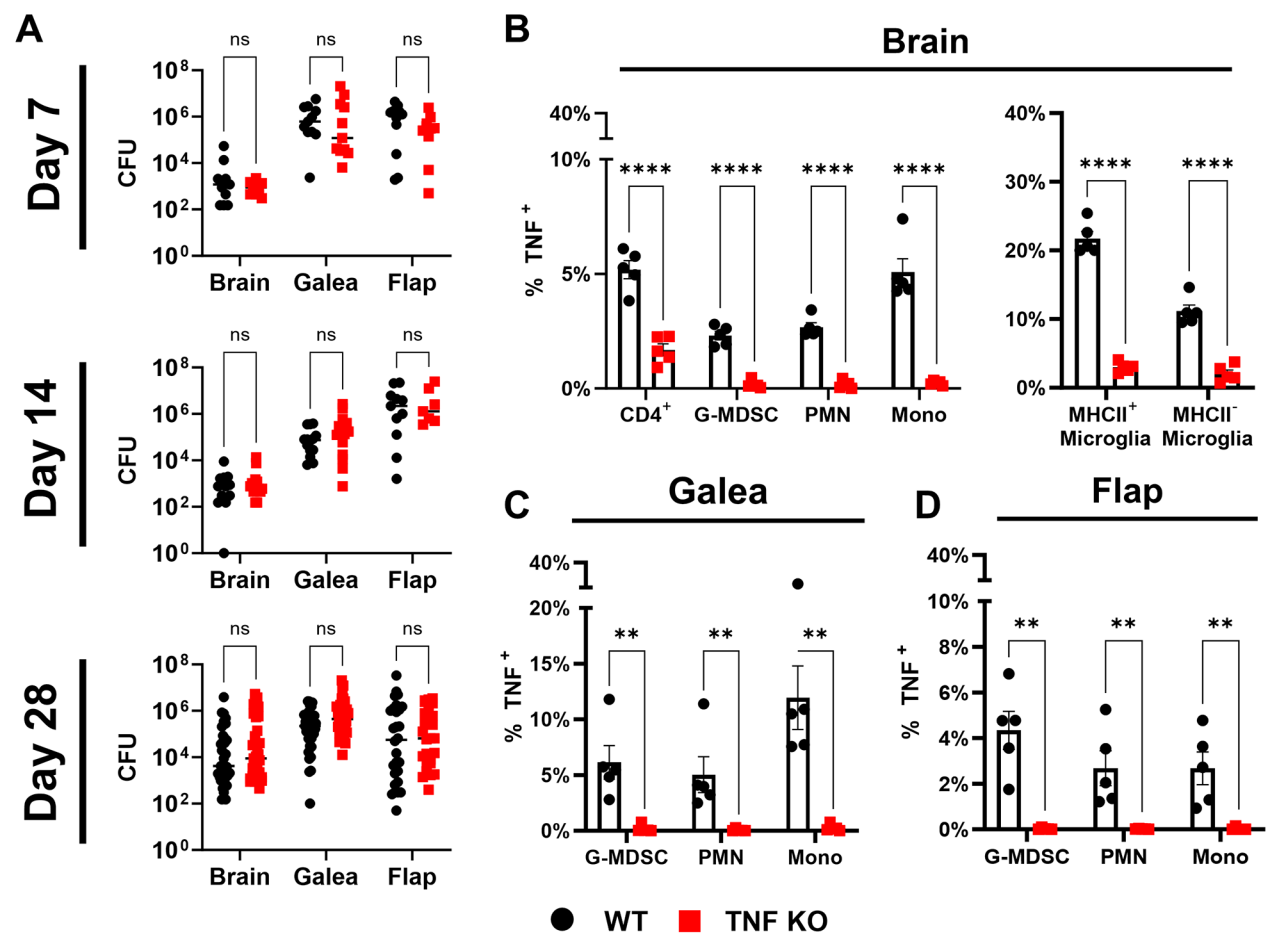
TNF does not impact bacterial burden during *S. aureus* craniotomy infection. TNF knockout (KO; *n* = 7–35) and wild type (WT; *n* = 11–35) mice were subjected to craniotomy infection and sacrificed at the indicated time points. (**A**) Bacterial burden was assessed at days 7, 14, and 28 post-infection (data was pooled from 2–5 independent experiments and analyzed by two-way ANOVA). (**B-D**) Intracellular staining for TNF was performed at day 7 post-infection in cells obtained from the brain (**B**), galea (**C**), and bone flap (**D**) (*n* = 5/group) to confirm the absence of TNF in KO cells (data are representative of two experiments (mean ± SEM) and analyzed by non-parametric multiple Mann-Whitney tests). CFU, colony forming unit; Mono, monocyte; **, *p* > 0.01; ****, *p* > 0.0001; ns, not significant

Our previous study identified a transcriptional footprint of TNF signaling during craniotomy infection, which was particularly elevated in granulocytes [8]. We have also reported robust TNF production by G-MDSCs, PMNs, macrophages, and microglia in vitro as well as in vivo during craniotomy infection [7]. Given these reports and the documented importance of TNF in immunity to other *S. aureus* infections [17, 23–26], we first explored how TNF influenced the immune response to *S. aureus* craniotomy infection using our mouse model [1, 2, 7–11] with TNF KO and WT animals. Dramatic reductions in leukocyte recruitment were observed in TNF KO mice in a time-dependent manner, where granulocytes were significantly decreased uniformly in the brain, galea, and bone flap of TNF KO mice at day 14 post-infection, both by relative abundance (Fig. 1D-F) and absolute count (Additional File 1: Fig. S2D-F). Interestingly, these deficiencies recovered to WT levels by day 28 post-infection (Fig. 1G-I; Additional File 1: Fig. S2G-I). At earlier intervals (i.e., day 7) no changes in relative immune cell abundance were observed between TNF KO and WT mice in the brain (Fig. 1A); however, by absolute cell counts, G-MDSCs, PMNs, monocytes, and NK cells were significantly decreased in TNF-deficient mice (Additional File 1: Fig. S2A). G-MDSCs in the galea and bone flap and PMNs in the galea were also modestly reduced in TNF KO mice at day 7 post-infection (Fig. 1B-C; Additional File 1: Fig. S2B-C). Intracellular cytokine staining confirmed the absence of TNF production in immune populations from the brain, galea, and bone flap of TNF KO animals (Fig. 2B-D), and sham craniotomy elicited few immune infiltrates (Additional File 1: Fig. S3A-D), directly implicating infection as the cause of leukocyte recruitment, as opposed to danger-associated molecular patterns released from damaged tissues. Despite the transient defects in leukocyte recruitment in TNF KO mice, bacterial burdens were similar between WT and TNF KO animals across the 28-day infection period (Fig. 2A) and antibiotic administration did not reveal additional changes in bacterial abundance between TNF KO and WT conditions (Additional File 1: Fig. S3E). These results illustrate the temporal importance of TNF for leukocyte recruitment but presumably not antimicrobial capabilities during craniotomy infection given the stability of bacterial growth, suggesting the existence of mechanisms that can compensate for TNF loss.

### TNF-dependent leukocyte recruitment is reflected by differential chemokine production

Given that granulocytes are the predominant cellular infiltrate in the galea and bone flap during craniotomy infection [1] and are in close proximity to the biofilm [2], we next explored the potential mechanism responsible for TNF-dependent granulocyte recruitment. We first assessed whether TNF loss affected BBB integrity to regulate leukocyte influx into the CNS during craniotomy infection. This was based on extensive literature showing that TNF production, particularly by microglia, regulates BBB properties to promote peripheral immune cell influx [1, 18, 44–46] and BBB permeability is reduced in TNF-deficient mice [47]. Albumin is a large plasma protein that is excluded from the CNS by an intact BBB [48] but accumulates during neuroinflammatory or pathologic conditions when the BBB is compromised [40, 49]. Albumin content in brain homogenates was equivalent between TNF KO and WT mice throughout the course of craniotomy infection (Additional File 1: Fig. S4A), indicating that differences in BBB permeability were not responsible for the transient reductions in leukocyte recruitment observed in TNF-deficient animals (Fig. 1).

**Fig. 3:**
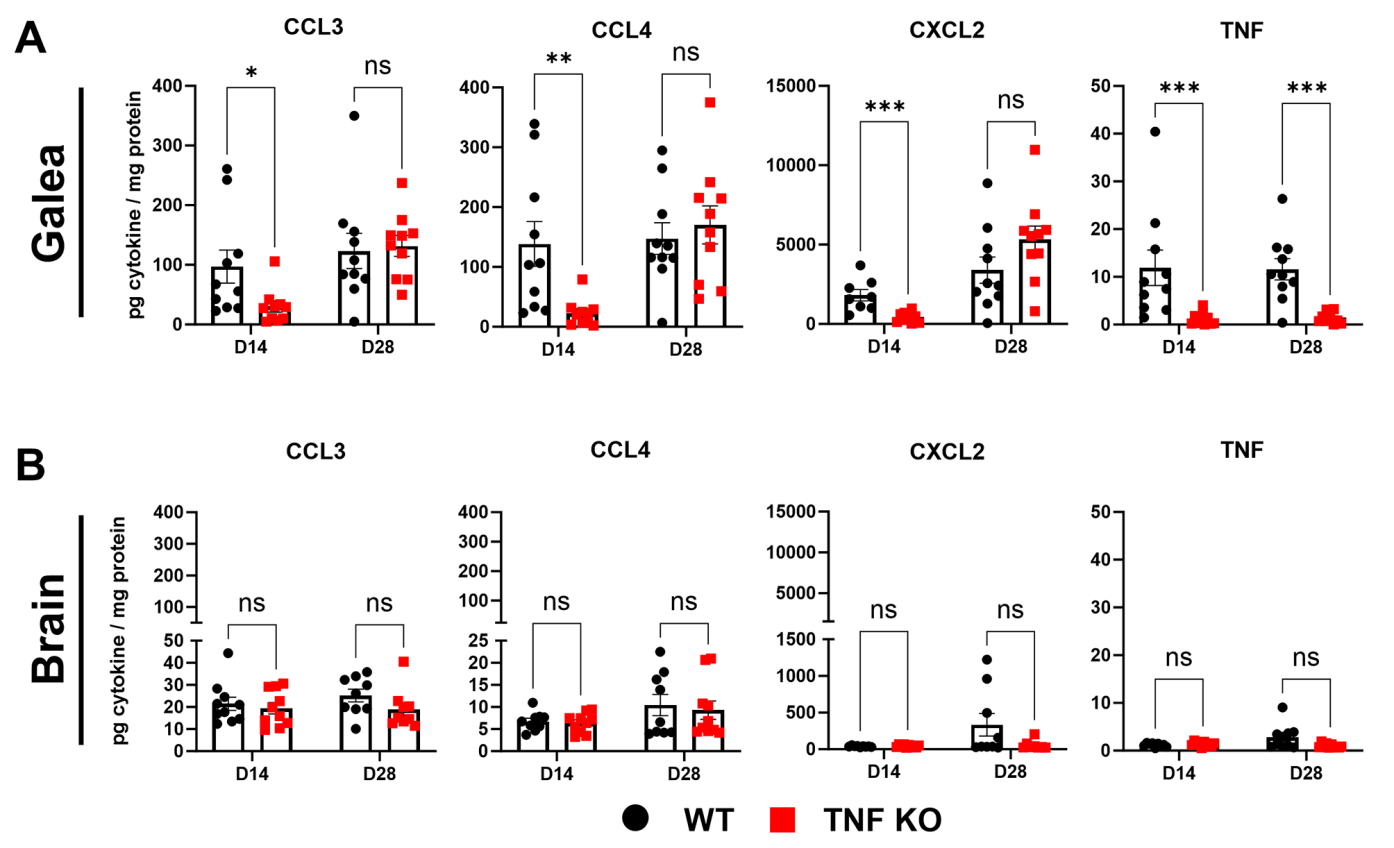
TNF influences the production of select chemokines during craniotomy infection. TNF knockout (KO; *n* = 8–10) and wild type (WT; *n* = 8–10) mice were subjected to craniotomy infection and sacrificed at the indicated timepoints, whereupon galea (**A**) and brain (**B**) tissues were homogenized, and inflammatory mediators were quantified from resulting supernatants. Data was pooled from 2 independent experiments (mean ± SEM) and analyzed by non-parametric multiple Mann-Whitney tests. *, *p* > 0.05; **, *p* > 0.01; ***, *p* > 0.001; ns, not significant

Among its pleiotropic effects, TNF is known to stimulate the production of other inflammatory mediators [50], including IL-6 [51], CCL3, CCL4, and CXCL2 [52]. To determine whether TNF loss impaired chemokine production to explain why leukocyte recruitment was reduced in TNF-deficient animals, a panel of 25 cytokines and chemokines was quantified in the galea and brain of TNF KO and WT mice at days 14 and 28 post-infection. TNF was detected in the galea of WT mice at both time points, with significant reductions observed in TNF KO animals (Fig. 3A). Significant decreases in CCL3, CCL4, and CXCL2 were observed in the galea of TNF KO mice at day 14 post-infection (Fig. 3A), corresponding to the timepoint and anatomical location where granulocyte recruitment was maximally reduced (Fig. 1E; Additional File 1: Fig. S2E). Further, these chemokines rebounded to WT levels at day 28 post-infection, mirroring the recovery of leukocyte infiltrates during craniotomy infection (Fig. 1H; Additional File 1: Fig. S2H). Other cytokines (Additional File 1: Fig. S4B) and chemokines (Additional File 1: Fig. S4C) in the brain were largely unaffected in TNF-deficient mice, likely due to the low expression of TNF in the brain of WT animals during craniotomy infection (Fig. 3B). IL-6 production was significantly reduced in TNF KO macrophages in response to live *S. aureus* (Additional File 1: Fig. S5A), and TNF was undetectable as expected (Additional File 1: Fig. S5D). In accordance with our *in* vivo findings (Additional File 1: Fig. S4B-C), TNF KO and WT macrophages produced largely equivalent amounts of CCL2 and IL-10 (Additional File 1: Fig. S5B-C). Overall, these results suggest that TNF is crucial for inducing chemokine and IL-6 production during craniotomy infection, specifically in the galeal compartment, aligning with previous reports [50–52].

### TNF-deficient granulocytes upregulate lymphotoxin-α during craniotomy infection

**Fig. 4:**
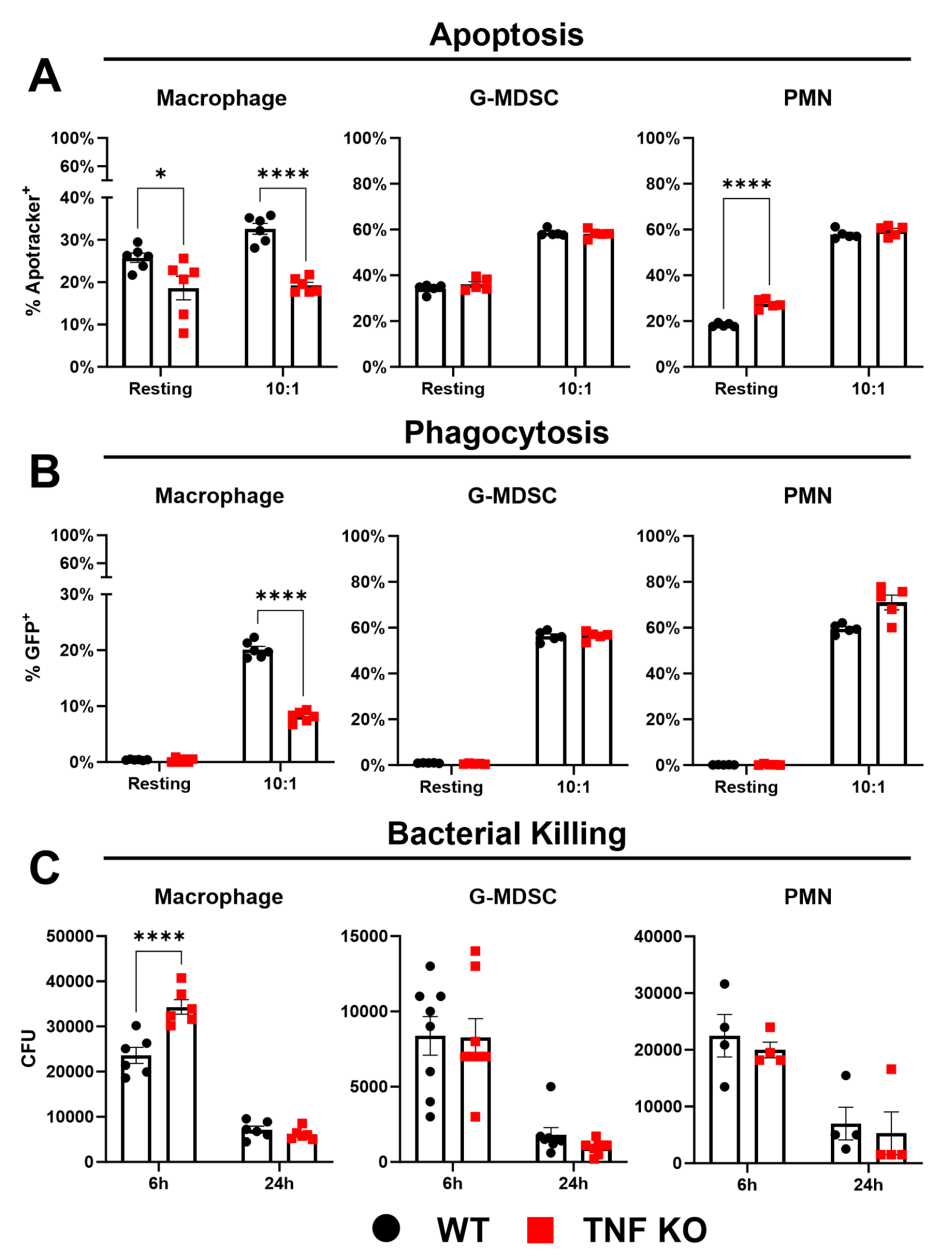
TNF modulates apoptosis, phagocytosis, and bactericidal activity in macrophages but not granulocytes. Primary leukocytes were challenged with live *S. aureus* at a MOI of 10:1 (bacteria: leukocyte), whereupon apoptosis (**A**) and phagocytosis (**B**) were quantified by flow cytometry (*n* = 5/group). Bacterial killing (**C**) was measured by gentamicin protection assays (*n* = 4–8/group). Data is representative of 2–3 independent experiments (mean ± SEM) and analyzed by two-way ANOVA. CFU, colony forming unit; *, *p* > 0.05; ****, *p* > 0.0001

While TNF influenced chemokine production and leukocyte recruitment to the galea during craniotomy infection (Figs. 1 and 3), these changes were not destabilizing enough to alter bacterial burden (Fig. 2A). This makes craniotomy infection unique from other *S. aureus* models where TNF is critical for bacterial containment [17, 23–26]. To explore why *S. aureus* titers were not affected following TNF loss, we first examined whether TNF influenced macrophage, PMN, and G-MDSC responses to *S. aureus*- the major cellular infiltrates during craniotomy infection [1]. Macrophage activity was selectively regulated by TNF, since apoptosis (Fig. 4A), phagocytosis (Fig. 4B), and bactericidal activity (Fig. 4C) were significantly reduced in TNF KO macrophages in response to live *S. aureus*, whereas granulocyte activation was largely unaffected by TNF loss (Fig. 4A-C). Examination of PMN bactericidal activity at earlier time points (2 h post-stimulation; Additional File 1: Fig. S6E) as well as apoptosis and cell death at later intervals (6 h and 24 h post-infection; Additional File 1: Fig. S6A-D) revealed only subtle differences in TNF KO vs. WT PMNs, which were unlikely to be biologically relevant based on their limited magnitude. Collectively, these experiments indicate that TNF is critical for macrophage antimicrobial capabilities and survival, in agreement with previously reported anti-apoptotic and proinflammatory effects of TNF signaling [12, 16, 50, 53].

**Fig. 5:**
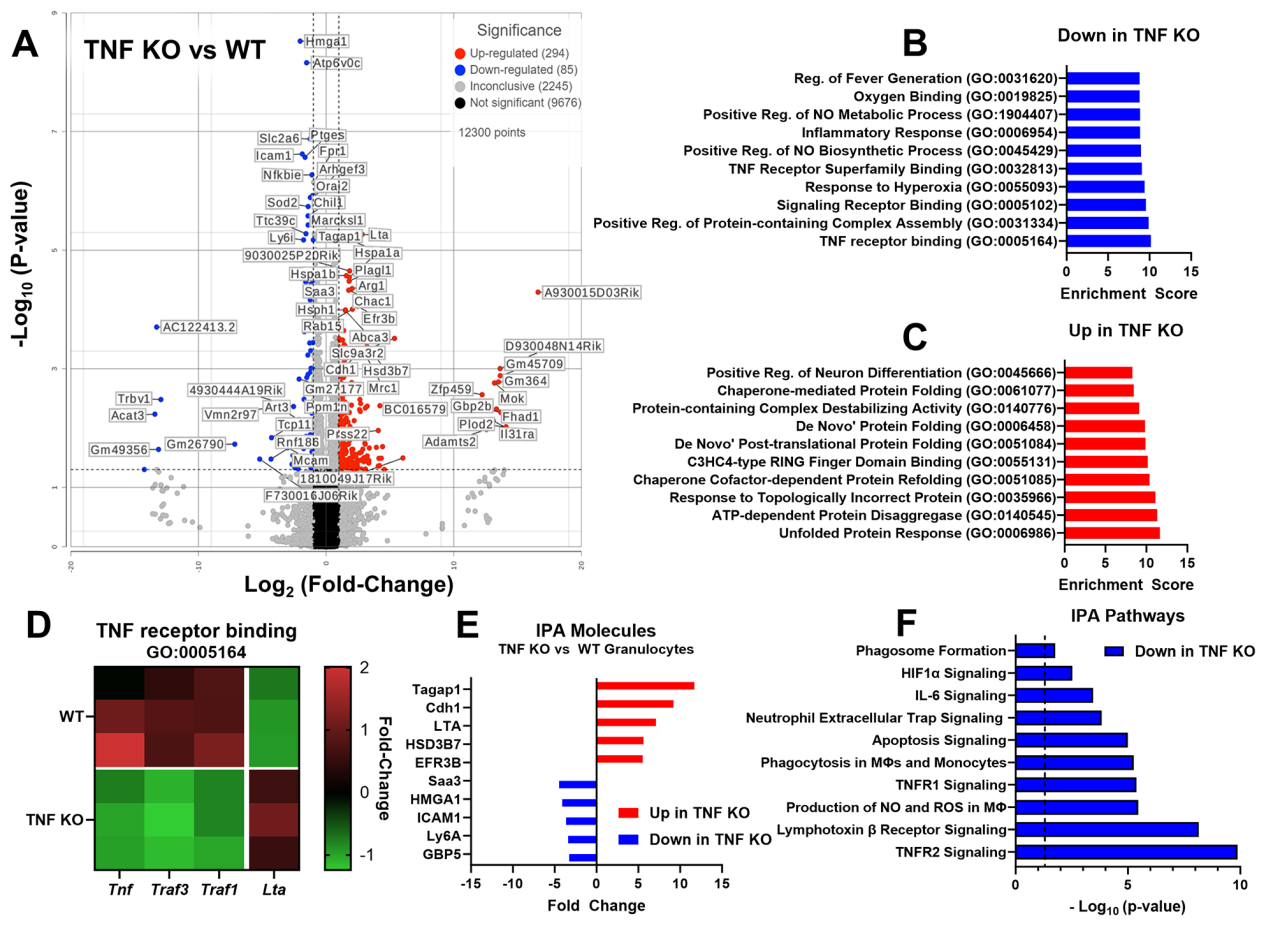
*Lta* expression is heightened in TNF knockout (KO) granulocytes during *S. aureus* craniotomy infection. TNF KO and WT mice were subjected to craniotomy infection and sacrificed on day 7 post-infection, whereupon live CD45^+^Ly6G^+^ granulocytes were isolated from galea tissues by FACS (*n* = 3 biological replicates, each pooled from 3 mice) and RNA isolated for bulk RNA-seq. (**A**) Volcano plot depicting genes significantly upregulated in WT (blue; 85 genes) or TNF KO (red; 294 genes) mice. Gene set enrichment analysis of pathways significantly decreased (**B**) or increased (**C**) in TNF KO granulocytes compared to WT. (**D**) Genes in the TNF receptor binding pathway (GO:0005164) were analyzed between TNF KO and WT granulocytes. (**E-F**) Ingenuity Pathway Analysis was conducted to predict differentially regulated molecules (**E**) and pathways (**F**). All reported transcriptional comparisons are statistically significant (*p* < 0.05)

Since TNF production was most robust in the galea, which is dominated by granulocyte infiltrates, one possibility to account for the lack of altered bacterial burden in TNF KO mice may be due to granulocyte insensitivity to TNF loss. However, TNF signaling has been reported to promote granulocyte function during *S. aureus* infection [17] and was transcriptionally enriched in granulocytes during craniotomy infection in WT mice [8]. Therefore, bulk RNA-seq was used to compare the transcriptional signatures of TNF KO and WT granulocytes to identify potential compensatory mechanisms in the context of TNF loss. Granulocytes (CD45^+^Ly6G^+^) were FACS purified from the galea of TNF KO and WT mice at day 7 post-infection for bulk RNA-seq. A total of 294 and 85 genes were upregulated in TNF KO vs. WT granulocytes, respectively (Fig. 5A). Gene set enrichment analysis confirmed that TNF signaling pathways were diminished with TNF loss (TNF Receptor Superfamily Binding, GO:0032813; TNF receptor binding, GO:0005164) concomitant with decreases in reactive oxygen/nitrogen species and inflammatory pathways (Fig. 5B). Similar findings were obtained using Ingenuity Pathway Analysis (IPA; Fig. 5F). In contrast, multiple pathways associated with protein misfolding/unfolded protein response (UPR) were enriched in TNF KO granulocytes (Fig. 5C). A more detailed examination of genes in the TNF receptor binding pathway confirmed decreased *Tnf*,* Traf3*, and *Traf1* levels in TNF KO granulocytes; however, lymphotoxin-α (*Lta*) expression was dramatically increased (Fig. 5D). In addition, IPA identified LTA (LTα) as the third most upregulated molecule in TNF KO cells relative to WT (Fig. 5E). Importantly, LTα shares 50% homology to TNF [54] and can signal through both TNFR1 and TNFR2 [55], possibly compensating for TNF deficiency in KO mice during craniotomy infection.

### TNF receptors have unique effects on leukocyte function but do not influence *S. aureus* craniotomy infection outcome

**Fig. 6:**
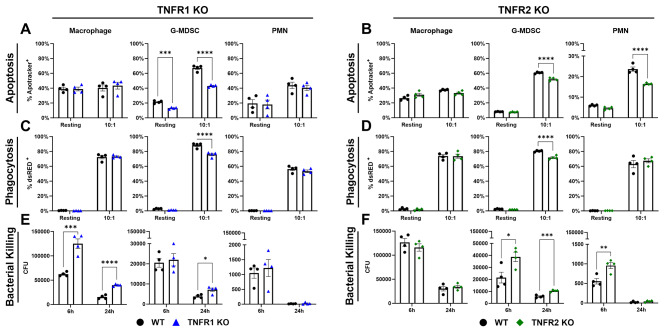
TNFR1 and TNFR2 differentially modulate macrophage and granulocyte apoptosis, phagocytosis, and bactericidal activity. Primary leukocytes from TNFR1 (**A**,** C**,** E**) or TNFR2 (**B**,** D**,** F**) KO mice were challenged with live *S. aureus* at a MOI of 10:1 (bacteria: leukocyte), whereupon apoptosis (**A-B**) and phagocytosis (**C-D**) were quantified by flow cytometry (*n* = 4–5/group). Bacterial killing (**E-F**) was measured by gentamicin protection assays (*n* = 4/group). Data is representative of 1–3 independent experiments (mean ± SEM) and analyzed by multiple unpaired t-test. CFU, colony forming unit; *, *p* > 0.05; **, *p* > 0.01; ***, *p* > 0.001; ****, *p* > 0.0001

Since TNF-deficient granulocytes augmented *Lta* expression, which can signal through both TNFR1 and TNFR2, we next investigated how the loss of either receptor regulated leukocyte function. A recent report revealed a role for TNF and its receptors in neutrophil extracellular trap (NET) formation [17]; however, no significant differences in NET formation were observed in vitro in TNF, TNFR1, or TNFR2 KO PMNs relative to WT cells (Additional File 1: Fig. S7A-I). Accordingly, in vivo experiments with mice incapable of forming NETs (PAD4 KO) [56] revealed no defects in bacterial containment (Additional File 1: Fig. S7J), suggesting that NET formation is dispensable during craniotomy infection. In contrast, TNFR1 and TNFR2 were found to differentially regulate leukocyte phagocytosis, bactericidal activity, and apoptosis in response to *S. aureus*. Both TNFR1 and TNFR2 deficiency decreased apoptosis in G-MDSCs (Fig. 6A-B), whereas PMN apoptosis was only affected by TNFR2 loss (Fig. 6B). Interestingly, macrophage apoptosis was unaffected by either TNFR1 or TNFR2 (Fig. 6A-B). Phagocytosis was modestly reduced in both TNFR1 and TNFR2 KO G-MDSCs, with all other cell types unaffected (Fig. 6C-D). Finally, TNFR1 was critical for promoting macrophage bactericidal activity (Fig. 6E), suggesting that similar observations in TNF KO macrophages (Fig. 4C) were likely TNFR1-dependent. Conversely, TNFR2 was important for bacterial containment by G-MDSCs and PMNs (Fig. 6F). However, since bactericidal activity was not affected in either TNF KO granulocyte population (Fig. 4C), this suggests that LTα may compensate for the loss of TNF in these cells via TNFR2 signaling. Collectively, these findings illustrate additional contributions of TNF signaling in granulocytes that only manifest in the context of receptor loss.

**Fig. 7:**
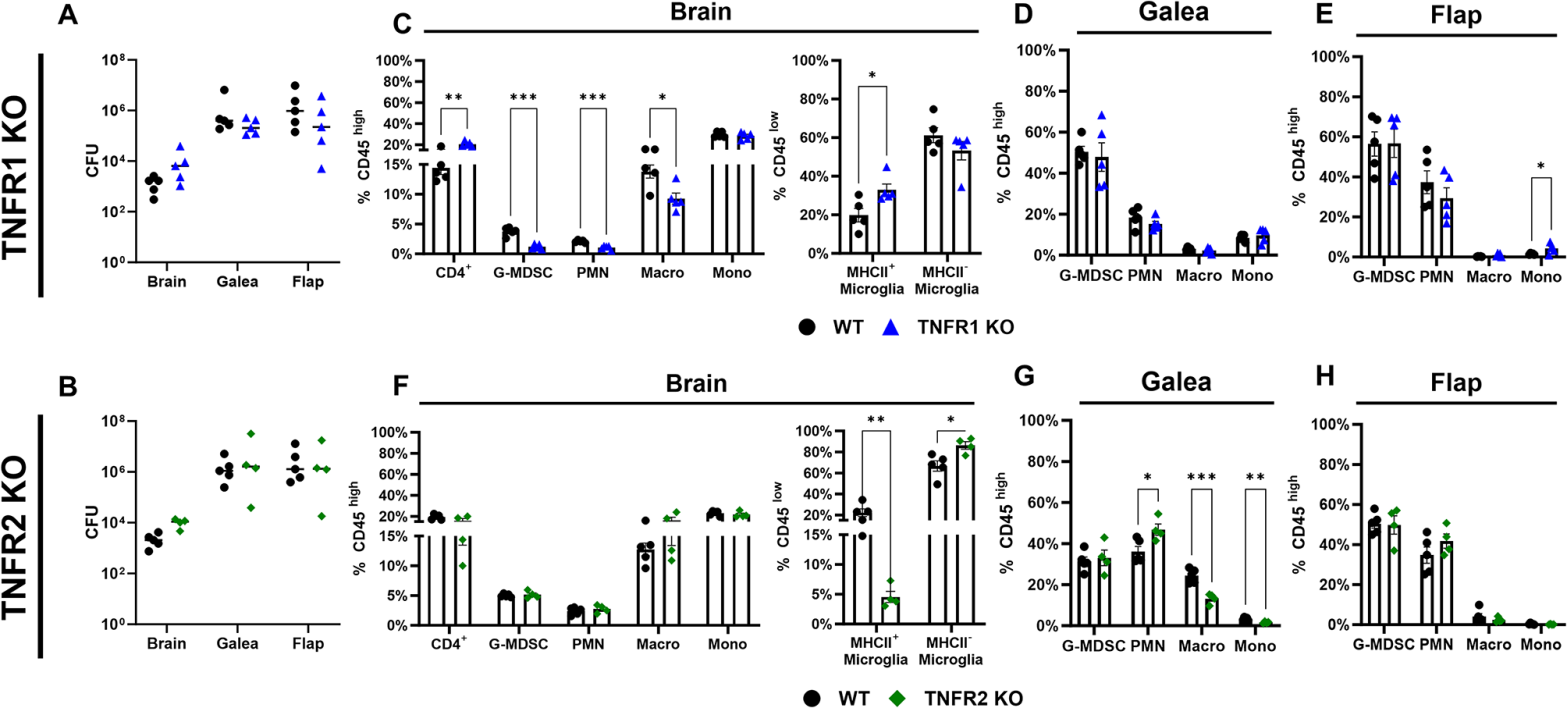
TNFRs do not impact bacterial burden during *S. aureus* craniotomy infection. TNFR1 (**A**,** C**,** D**,** E**) or TNFR2 (**B**,** F**,** G**,** H**) knockout (KO; *n* = 4–5) and wild type (WT; *n* = 5) mice were subjected to craniotomy infection and sacrificed at day 7 post-infection, whereupon bacterial burden (**A-B**) and immune populations (**C-E**,** F-H**) were assessed. Results were analyzed by (**A-B**) two-way ANOVA and (**C-E**,** F-H**) multiple unpaired t-test (mean ± SEM). CFU, colony forming units; Macro, macrophage; Mono, monocyte; *, *p* > 0.05; **, *p* > 0.01; ***, *p* > 0.001

Given these results, we next investigated whether TNFR1 and/or TNFR2 signaling influenced *S. aureus* craniotomy infection. The phenotype of TNFR1 KO mice was partially reminiscent of TNF KO animals, as revealed by significant reductions in G-MDSC, PMN, and macrophage infiltrates in the brains of TNFR1 KO mice with no change in bacterial burden (Fig. 7A and C). However, leukocyte influx was not altered in either the galea or bone flap (Fig. 7D and E). TNFR2 loss led to significant decreases in MHCII^+^ activated microglia in the brain (Fig. 7F) and the galea displayed minor alterations in leukocyte recruitment (Fig. 7G), although this was not sufficient to influence bacterial burdens in either compartment (Fig. 7B). In general, similar trends were observed for absolute leukocyte counts in infected TNFR1 and TNFR2 KO animals (Additional File 1: Fig. S88). Collectively, while granulocytes augment *Lta* expression in the context of TNF deficiency, the fact that bacterial burdens were not dramatically altered in TNF, TNFR1, or TNFR2 KO mice indicates that TNF signaling in isolation is not sufficient to alter the course of craniotomy infection.

## Discussion

This study demonstrates the importance of TNF signaling for leukocyte recruitment during *S. aureus* craniotomy infection, where dramatic reductions in cellular influx were observed in the brain, galea, and bone flap of TNF KO mice during the first two weeks post-infection. Interestingly, these changes largely returned to WT levels by day 28, underscoring the temporal complexity of the immune response during craniotomy infection. Fluctuations in leukocyte recruitment coincided with concurrent decreases and recovery in the levels of several granulocyte-recruiting chemokines, including CCL3, CCL4, and CXCL2 in TNF KO mice, likely accounting for reduced PMN and G-MDSC abundance at earlier time points. Interestingly, despite impaired leukocyte recruitment up to day 14 post-infection in TNF KO animals, this did not translate to altered bacterial burden in any infection compartment analyzed. This finding contrasts with prior reports of heightened bacterial growth or even lethality with TNF deficiency in other models of *S. aureus* infection [17, 23–26] or alternative pathogens [52, 57–60]. Our RNA-seq studies revealed heightened *Lta* expression in TNF KO granulocytes, which led us to speculate that LTα may compensate to protect TNF KO mice from exaggerated pathology since it binds the same receptors as TNF (i.e., TNFR1 and TNFR2) to exert downstream effects. However, this possibility was not supported by subsequent experiments utilizing TNFR1 and TNFR2 KO mice. Instead, this suggests that other factors unique to the craniotomy infection microenvironment influence the TNF-independent phenotypes reported here. One possibility relates to the nature of *S. aureus* growth during craniotomy infection, namely biofilm formation on the infected skull bone [1, 2, 10, 11]. Biofilm infections are chronic [61] and thus often exhibit divergent attributes from other bacterial infections that are typified by planktonic growth (i.e., abscesses, sepsis) [1, 2, 10, 31, 34, 62–64]. To our knowledge, only one study reported heightened infectious burden in TNF KO mice using a biofilm model (orthopedic biofilm infection) [26], which described only modest increases in bacterial growth. In contrast, work with non-biofilm infection models often revealed more profound increases in pathogen burden, sometimes resulting in lethality [17, 23–25, 52, 57–60]. Therefore, TNF may be less critical for an effective antibacterial response in the context of biofilm infection. This dichotomy between the importance of TNF across planktonic and biofilm infections is supported by our in vitro data, where TNF deficiency decreased macrophage phagocytosis and bactericidal activity in response to planktonic *S. aureus*, which diverged from our in vivo results showing equivalent bacterial burden during TNF KO or WT craniotomy infection that is typified by biofilm formation. Other in vivo data presented here also supports this concept, where NET formation had no impact on *S. aureus* craniotomy infection. However, NET formation was found to be protective in a mouse model of *S. aureus* skin and soft tissue infection, reflective of planktonic growth, by TNF signaling through TNFR2 [17]. Therefore, the similarities in bacterial burden between WT and TNF, TNFR1, and TNFR2 KO mice observed in this study support the ineffectiveness of TNF-induced NETs to combat biofilm infection. This earlier report [17] identified that TNFR1 was responsible for granulocyte recruitment, which agrees with our findings where the greatest reductions in PMN and G-MDSC infiltrates were observed in the brains of TNFR1 compared to TNFR2 KO mice. Nevertheless, TNFR1 and TNFR2 deficiency did not affect bacterial burden in vivo, despite phenotypic changes in granulocyte phagocytosis, apoptosis, and bactericidal activity in vitro, reflecting the involvement of additional factors in the biofilm inflammatory milieu that promote infection persistence, which remain to be identified. Given our recent findings that immune responses during human craniotomy infection are highly conserved with the mouse model at the cellular and transcriptional levels (manuscript in revision) and despite the reported association between anti-TNF biologic use and an increased risk of *S. aureus* infection [22], our findings suggest that the biofilm features of *S. aureus* during craniotomy infection may circumvent a negative outcome in this regard, although this remains highly speculative. However, it is important to note that this line of reasoning may not extend to other causative pathogens of craniotomy infection [65], which could be exacerbated by TNF depletion and remains to be assessed.

Another concept underscored by this work surrounds the biological redundancy of TNF signaling, which is a complex system deeply integrated with cellular function [50, 55]. We identified a novel increase in *Lta* expression in TNF KO granulocytes, suggesting a potential compensatory response since LTα can signal through both TNFR1 and TNFR2 [54]. This was supported by the finding that apoptosis, phagocytosis, and bactericidal activity were similar in PMNs from TNF KO and WT mice, whereas significant deficits were observed for these same measures under TNFR2 and, to a lesser extent, TNFR1 deficiency. These findings demonstrate that TNF deletion may cause unexpected transcriptional changes that could mask or alter phenotypes. In addition to *Lta*, our analysis revealed a marked increase in pathways associated with misfolded proteins/UPR in TNF KO granulocytes. This finding was surprising, as TNF has been reported to induce the UPR [66–68] and, therefore, was expected to be reduced with TNF deficiency. However, the UPR has also been shown to promote TNF production by immune cells [69, 70], so this may reflect a compensatory effort by granulocytes to increase TNF levels, although this was futile in the context of *Tnf* deletion. Another footprint of TNF action in vivo was decreased PMN and G-MDSC recruitment in all three tissue compartments of TNF KO animals, which peaked at day 14 and recovered by day 28 post-craniotomy infection. This phenotype was linked to reduced chemokine production, which paralleled trends in cellular abundance. Interestingly, an earlier study reported nearly identical changes in a model of tuberculosis (TB) infection with TNF KO mice [52], in agreement with our findings. For example, leukocyte recruitment to the liver and spleen was impaired in TB-infected TNF KO mice up to day 14 post-infection, with recovery to (and sometimes exceeding) WT levels at day 28, identical to the time frames presented in the current study. Additionally, CCL3, CCL4, and CXCL2 levels were low at day 14 and recovered by day 28 post-infection, also in agreement with our findings [52]. The striking parallels between the report from Roach et al. and our data, despite differing pathogens, infection sites, models, and methodologies, suggest that the relationship between TNF, chemokines, and leukocyte recruitment is partially cell-intrinsic and not mediated by microenvironmental factors. While Roach et al. did not explore the mechanism responsible for TNF effects during TB infection, our data may provide some insights. Namely, heightened LTα production in the context of TNF deficiency may be sufficient over extended periods to stimulate CCL3, CCL4, and CXCL2 expression. However, because TNF had no impact on bacterial burden during craniotomy infection, this model is suboptimal to pursue this idea further. Additional work investigating the role of LTα in the presence and absence of TNF deficiency in a TNF-dependent model (such as TB infection) would be required to explore this possibility. Interestingly, progranulin (PGRN) and TNF also share the same receptor, and PGRN has a stronger affinity for TNFR than TNF [71]. In addition, PGRN is an important neurotrophic factor and plays an important role in infection, inflammation, and immunity [72–75]. Although *grn* expression was low and not significantly different between WT and TNF KO granulocytes during craniotomy infection, it is possible that other cell types are a source of PGRN, which could be pursued in future studies that are outside the scope of the current report. Additionally, although G-MDSCs exhibited modest bactericidal activity in vitro, it is unclear whether this would extend in vivo given the documented anti-inflammatory characteristics of G-MDSCs within the complex craniotomy infection microenvironment [9, 10, 76].

In conclusion, we demonstrate a role for TNF in leukocyte recruitment to the site of craniotomy infection by its ability to promote chemokine production. Additionally, we present evidence for a potential compensatory mechanism for the lack of TNF via enhanced *Lta* transcription. Despite these changes, infection was not worsened by TNF loss at any time point examined, which may result from *S. aureus*-derived factors unique to biofilm growth. These findings underscore the pleiotropic effects of TNF during bacterial infection and the heterogeneity in immune responses that are likely tissue niche-dependent.

### Electronic supplementary material

Below is the link to the electronic supplementary material.

Supplementary Material 1

## Data Availability

Transcriptomic data from bulk RNA-seq studies has been deposited in the GEO database (GSE252481). All other data in this manuscript are available upon reasonable request.

## Abbreviations

BCA: Bicinchoninic acid assay
CCL3: Chemokine (C–C motif) ligand 3 (MIP–1α)
CCL4: Chemokine (C–C motif) ligand 4 (MIP–1β)
CD3: cluster of differentiation 3
CD4: cluster of differentiation 4
CD45: cluster of differentiation 45
CD8: cluster of differentiation 8
CFU: Colony forming unit
CNS: Central nervous system
CO_2_: Carbon dioxide
CX3CR1: CX3C motif chemokine receptor 1
CXCL2: Chemokine (C–X–C motif) ligand 2 (MIP–2)
ELISA: Enzyme–linked immunosorbent assay
FACS: Fluorescence–activated cell sorting
FBS: Fetal bovine serum
GEO: Gene expression omnibus
GFP: Green fluorescent protein
G-CSF: Granulocyte colony stimulating factor
GM-CSF: Granulocyte-macrophage colony stimulating factor
IL-6: Interleukin-6
IPA: Ingenuity pathway analysis
KO: Knockout
LTα: Lymphotoxin–α
MAPK: Mitogen–activated protein kinase
G-MDSC: Granulocytic myeloid-derived suppressor cell
MHCII: Major histocompatibility complex II
MOI: Multiplicity of infection
NET: Neutrophil extracellular trap
NF-κB: Nuclear factor-kappa B
PBS: Phosphate–buffered saline
PGRN: Progranulin
PMN: Neutrophil
RNA: Ribonucleic acid
TB: Tuberculosis
TNF: Tumor necrosis factor
TNFR1: Tumor necrosis factor receptor 1
TNFR2: Tumor necrosis factor receptor 2
UPR: Unfolded protein response
WT: Wild type
