# Supplementary material for: Tumor necrosis factor regulates leukocyte recruitment but not bacterial persistence during Staphylococcus aureus craniotomy infection
Source: J Neuroinflammation. 2024 Jul 23;21:179. doi: 10.1186/s12974-024-03174-9 (PMC11264501; doi:10.1186/s12974-024-03174-9)
Supplement: Supplementary file 1 — Supplementary Material 1 [file 12974_2024_3174_MOESM1_ESM.pdf]

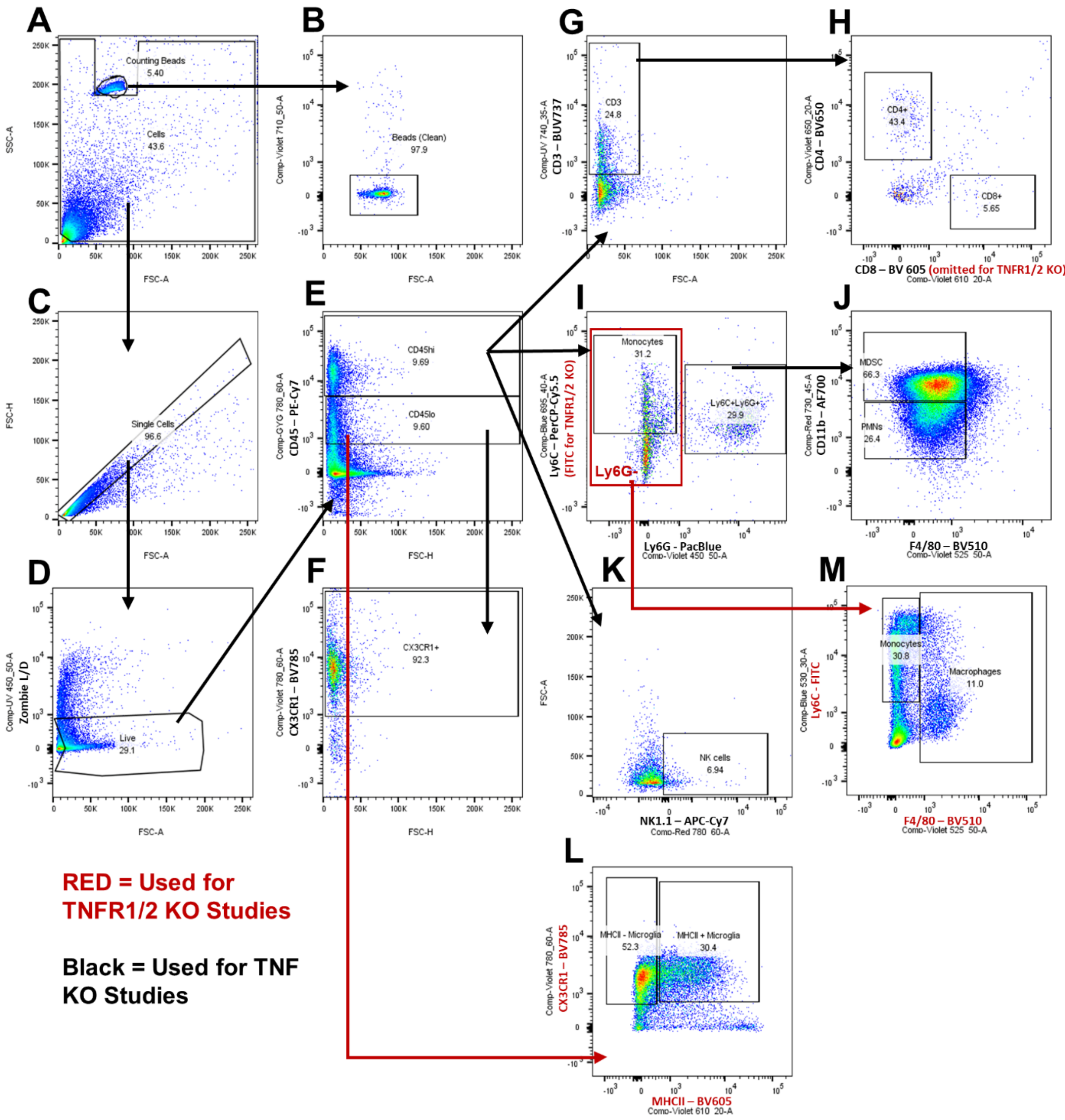


**Figure S1. Gating strategies for *in vivo* flow cytometry experiments.** From total events (**A**), counting beads were excluded (**B**) to quantify total cell counts and the remaining events were gated on FSC and SSC to isolate single (**C**) and subsequently live cells (**D**). Immune cells were then divided into CD45$^{low}$ and CD45$^{high}$ populations (**E**). CD45$^{low}$ cells (**E**) were further separated into microglia based on CXCR1 (**F**) for TNF KO studies and segregated further by

CXCR1/MHCII microglia (**L**) for TNFR1 and 2 KO experiments. CD45$^{high}$ cells (**E**) were gated on CD3 (**G**) and CD4/CD8 (**H**) to characterize T cells, Ly6C and Ly6G to separate granulocytes from monocytes (**I**), and NK1.1 to identify NK cells (**K**). Ly6G$^{+}$ cells (I) were further separated into neutrophils and G-MDSCs based on CD11b and F4/80 levels (**J**). Ly6G$^{-}$ cells were separated into monocytes and macrophages by Ly6C and F4/80 expression (**M**).

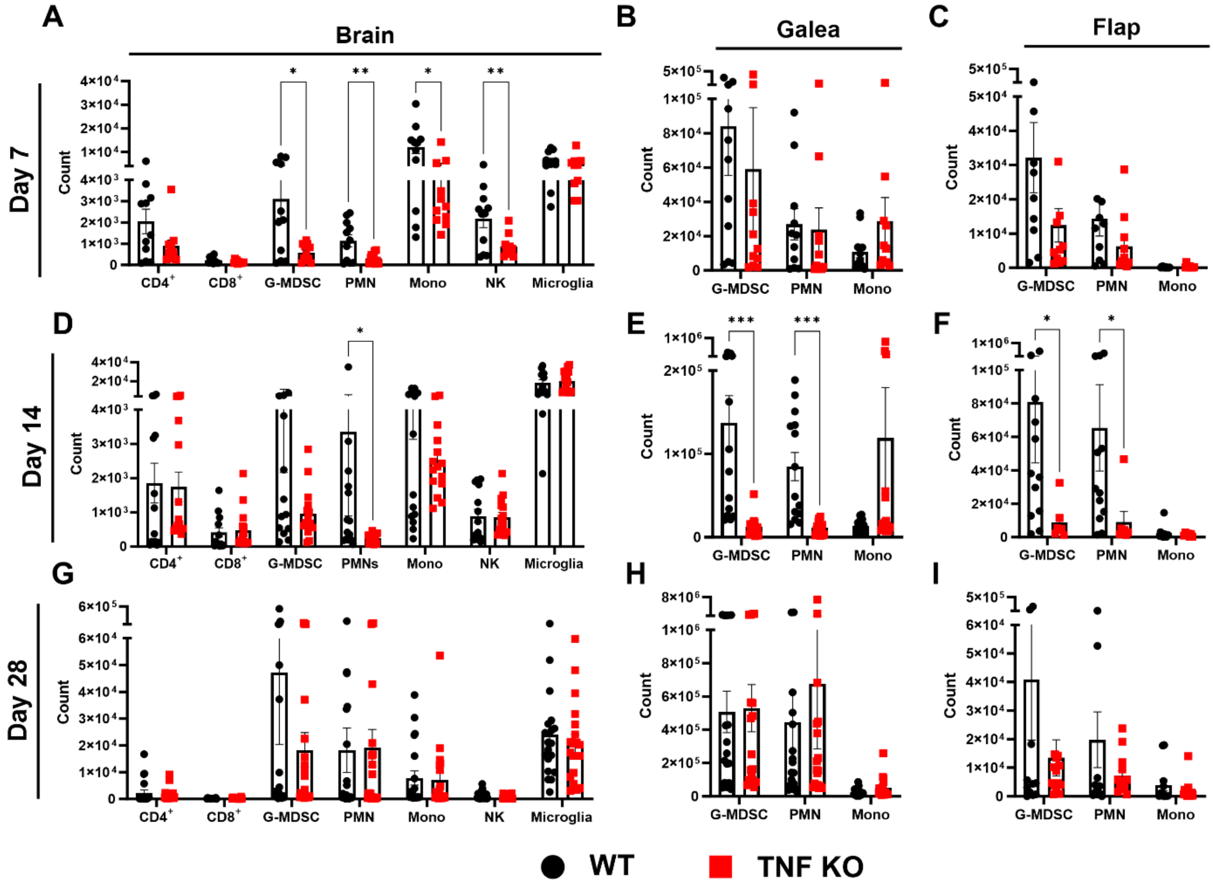


**Figure S2. Absolute cell counts of immune populations in TNF KO mice during *S. aureus* craniotomy infection.** TNF knockout (KO; n=7-35) and wild type (WT; n=11-35) mice were subjected to craniotomy infection and sacrificed at days 7 (**A-C**), 14 (**D-F**), and 28 (**G-I**) post-infection, whereupon absolute counts of immune populations were quantified in the brain (**A, D, G**), galea (**B, E, H**), and bone flap (**C, F, I**) using counting beads. Data was pooled from 2-5 independent experiments (mean ± SEM) and analyzed by multiple unpaired t-test. Mono, monocyte; *, $p$>0.05; **, $p$>0.01; ***, $p$>0.001.

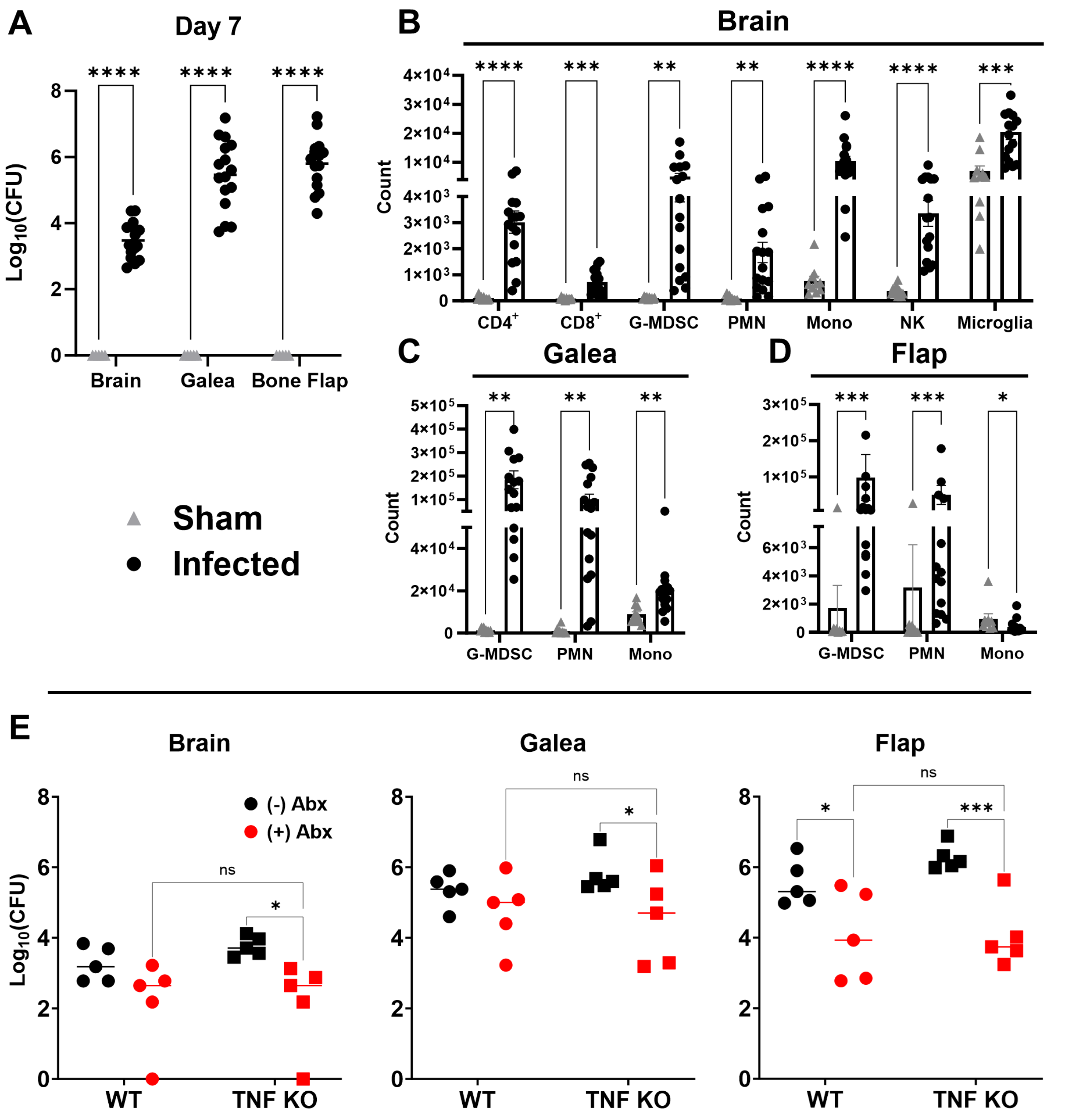


**Figure S3. Craniotomy infection actively elicits leukocyte recruitment and the lack of TNF involvement on bacterial burden is not altered with systemic antibiotics. (A-D)** Wild type (WT; n=15) mice were subjected to either sterile (sham) craniotomy or *S. aureus* infection and sacrificed at day 7 post-surgery, whereupon bacterial burden (**A**) and immune populations (**B-D**) were assessed as absolute counts. (**E**) WT and TNF KO mice were subjected to craniotomy infection ± daily antibiotic administration beginning at day 3 post-infection (n=5/group). Animals

were sacrificed at day 7 post-infection, whereupon bacterial burden in the brain, galea, and bone flap was assessed. Data analyzed by (**A**) 2-way ANOVA and (**B-D**) multiple unpaired t-test. Mono, monocyte; NK, NK cell; Abx, antibiotics; *, $p>0.05$; **, $p>0.01$; ***, $p>0.001$; ****, $p>0.0001$; ns, not significant.

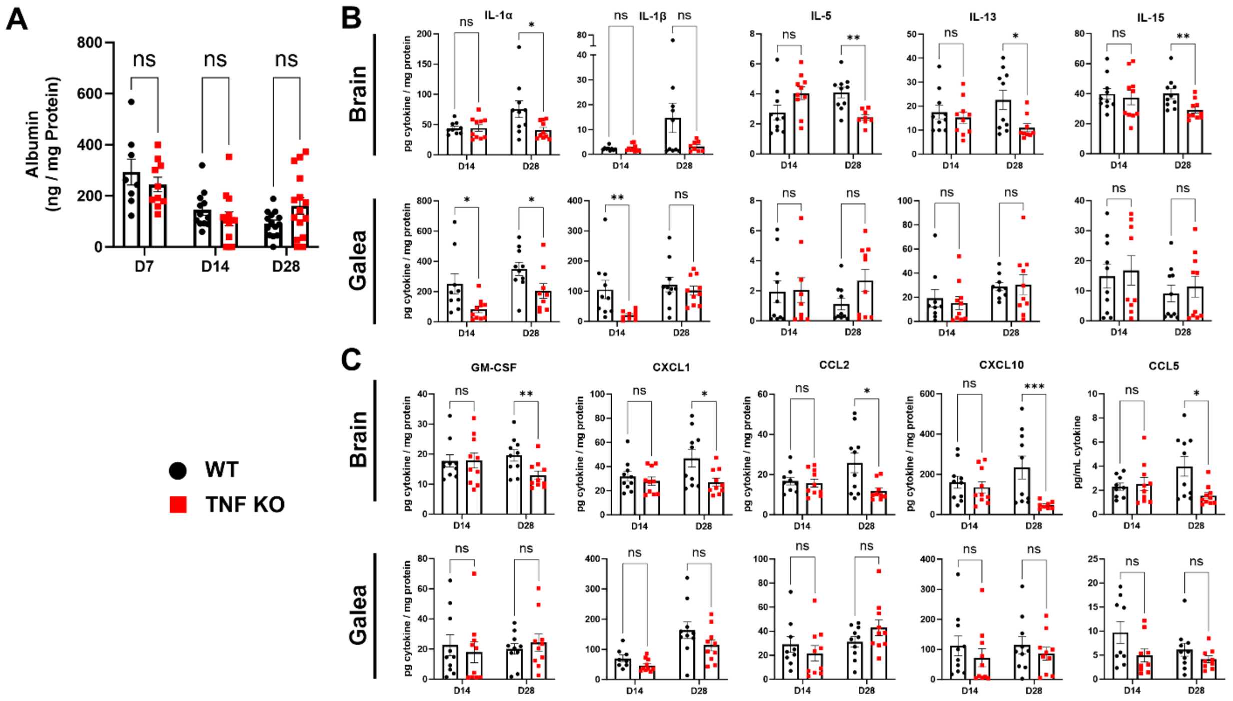


**Figure S4. Blood-brain barrier permeability during craniotomy infection is not altered following TNF loss.** TNF knockout (KO; n=10) and wild type (WT; n=10) mice were subjected to craniotomy infection and sacrificed at the indicated timepoints, whereupon (**A**) albumin levels were quantified in brain supernatants, and cytokines (**B**) and chemokines (**C**) were quantified in galea and brain homogenates. All measurements were normalized to total protein levels and pooled from 2 independent experiments (mean ± SEM). Data were analyzed by two-tailed Student's t-test (**A**) or non-parametric multiple Mann-Whitney test (**B**). *, $p$>0.05; **, $p$>0.01; ns, not significant.

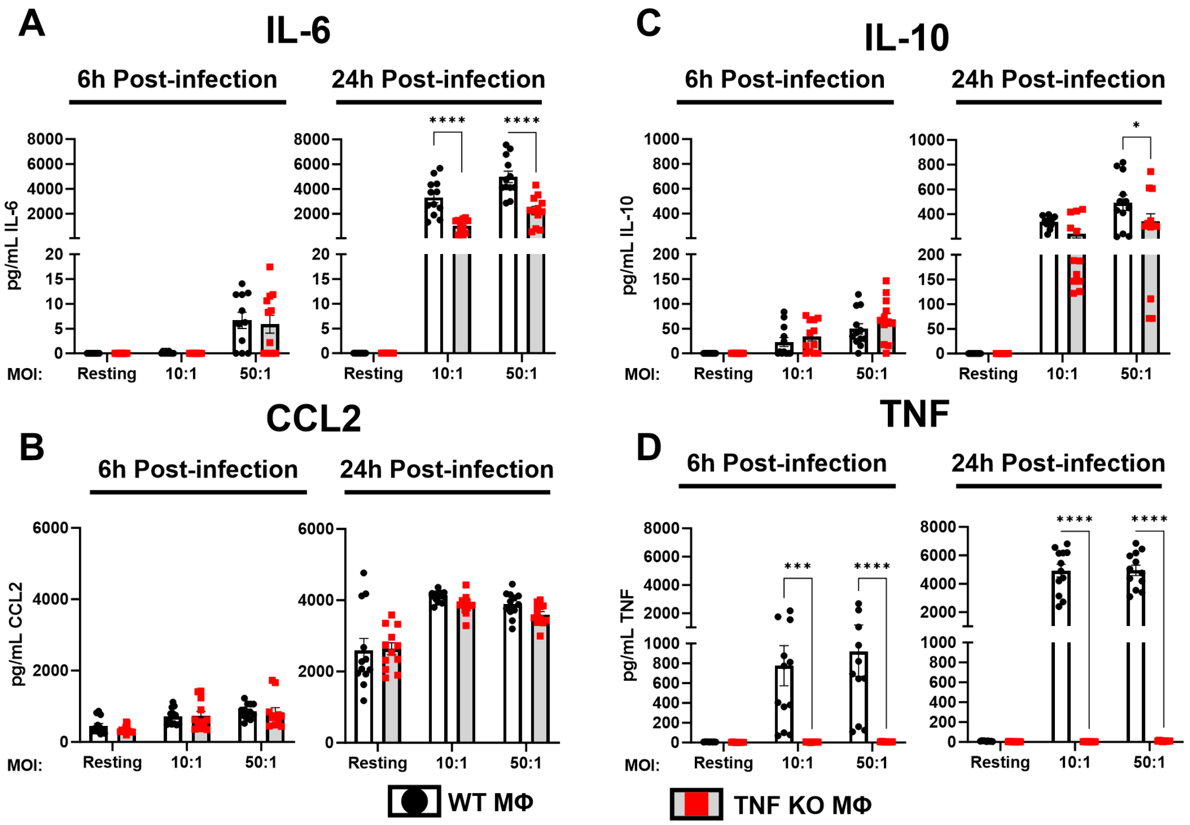


**Figure S5. TNF deficiency attenuates macrophage IL-6 production following *S. aureus* exposure.** Primary mouse macrophages (MΦs) were generated from wild type (WT; n=12) or TNF knockout (KO; n=12) mice and challenged with live *S. aureus* at a MOI of 10:1 or 50:1 for 1 h before elimination of extracellular bacteria by gentamicin treatment. Following incubation for the indicated duration, cell-conditioned supernatants were collected and assessed for (**A**) IL-6, (**B**) CCL2, (**C**) IL-10, and (**D**) TNF. Data combined from 3 independent experiments and analyzed by two-way ANOVA. *, $p>0.05$; ***, $p>0.001$; ****, $p>0.0001$.

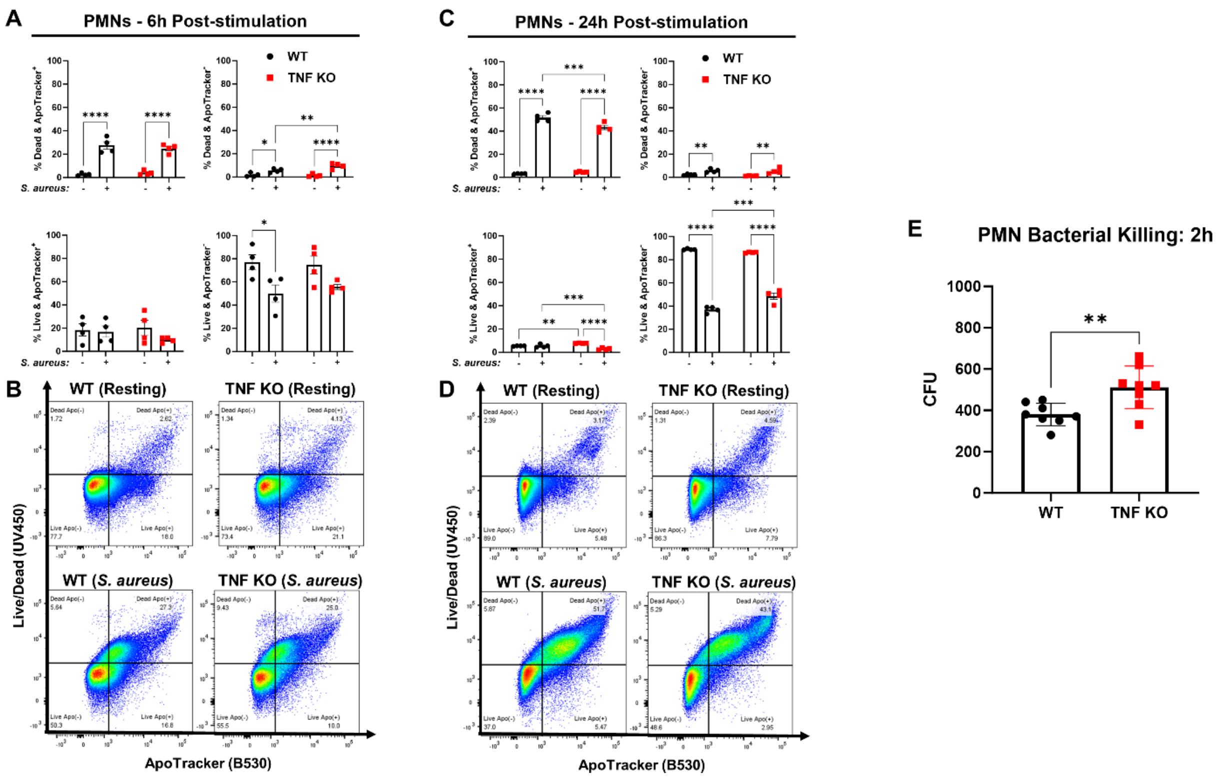


**Figure S6. Role of TNF on PMN viability, apoptosis, and bactericidal activity in response to *S. aureus*. (A-D)** Primary neutrophils (PMNs) from TNF knockout (KO; n=5) and wild type (WT; n=5) mice were challenged with live *S. aureus* at an MOI of 10:1 for 1h before elimination of extracellular bacteria with gentamicin. Following incubation for 6 (**A-B**) or 24 h (**C-D**), PMN viability and apoptosis was assessed by two-way ANOVA (**A, C**) with representative flow plots shown (**B, D**). (**E**) Primary PMNs from TNF KO (n=8) and WT (n=8) mice were stimulated with live *S. aureus* at an MOI of 10:1, whereupon bacterial killing was assessed at 2 h post-infection via gentamicin protection assay. Data analyzed by two-tailed t-test. *, *p*>0.05; **, *p*>0.01; ***, *p*>0.001; ****, *p*>0.0001.

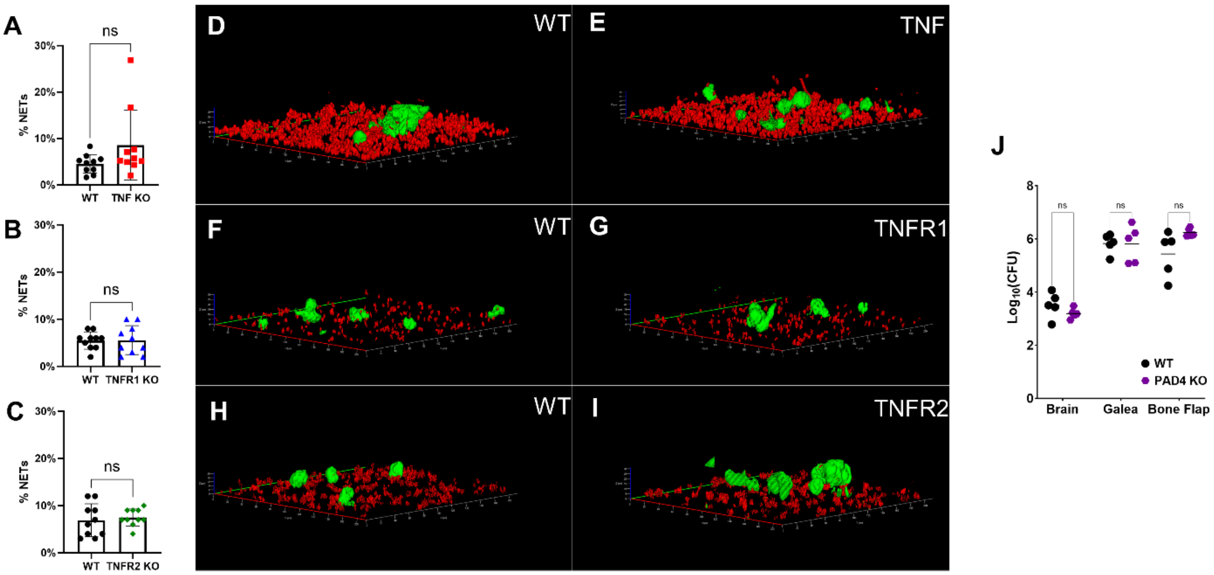


**Figure S7. TNF does not affect NET formation in response to *S. aureus*.** Primary PMNs from TNF (**A, E**), TNFR1 (**B, G**), and TNFR2 (**C, I**) knockout (KO; n=10) or wild type (WT; n=10) mice (**D, F, H**), were challenged with live *S. aureus*-dsRED (red) at a MOI of 10:1 for 1 h, and subsequently stained with Sytox Green (Green). NETs were quantified by the percentage of total cells in each 40x field of view (**A-C**). Representative Z-stack images from each condition are also shown (**D-I**). (**J**) WT and PAD4 KO mice (n=5/group) were subjected to *S. aureus* craniotomy infection and sacrificed at day 7 post-infection, whereupon bacterial burden was assessed from the brain, galea, and bone flap. Data analyzed by two-tailed Student's t-test (**A-C**) or 2-way ANOVA (**J**). ns, not significant.

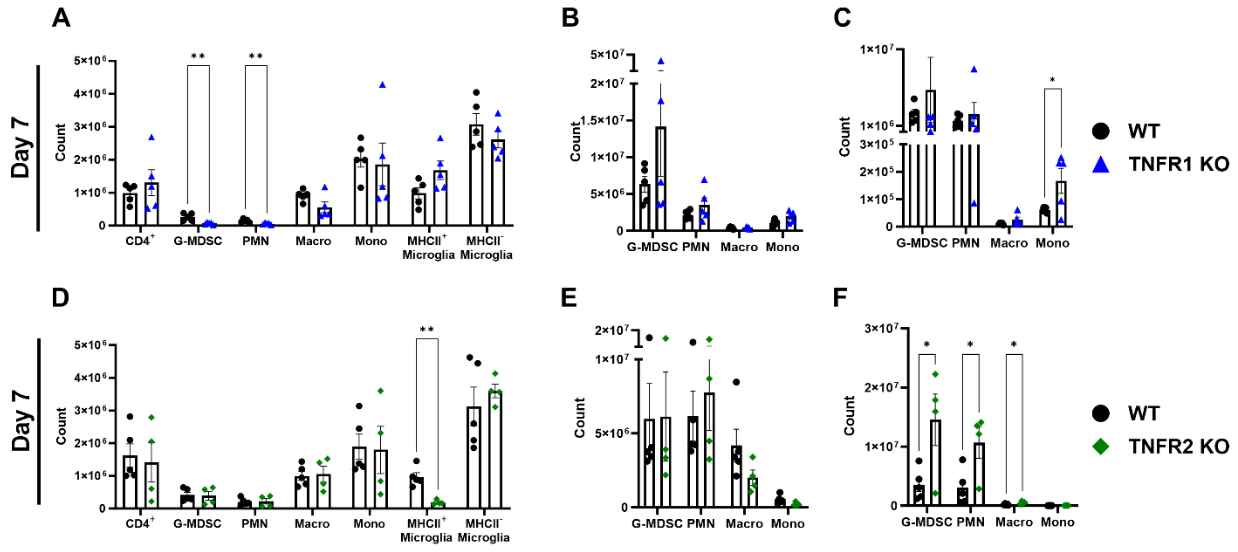


**Figure S8. Absolute cell counts of immune populations in TNFR1 or TNFR2 KO mice during *S. aureus* craniotomy infection.** TNFR1 (**A-C**) or TNFR2 (**D-F**) knockout (KO; n=4-5) and wild type (WT; n=5) mice were subjected to craniotomy infection and sacrificed at day 7 post-infection, whereupon absolute counts of immune populations in the brain (**A, D**), galea (**B, E**), and bone flap (**C, F**) were quantified using counting beads. Data was analyzed by multiple unpaired t-test (mean ± SEM). Macro, macrophage; Mono, monocyte; *, $p$>0.05; **, $p$>0.01.
